# NALP3-Inflammasome-Related Gene Polymorphisms in Patients with Prehypertension and Coronary Atherosclerosis

**DOI:** 10.1155/2016/7395627

**Published:** 2016-06-30

**Authors:** Xin Zhao, Chonghuai Gu, Chenghui Yan, Xiaolin Zhang, Yi Li, Li Wang, Lili Ren, Yan Zhang, Junyin Peng, Zhiming Zhu, Yaling Han

**Affiliations:** ^1^Cardiovascular Research Institute and Department of Cardiology, General Hospital of Shenyang Military Region, 83 Wenhua Road, Shenyang, Liaoning 110840, China; ^2^Jinzhou Medical University, Jinzhou 121000, China; ^3^Chongqing Institute of Hypertension, Daping Hospital, Third Military Medical University, Chongqing 404100, China

## Abstract

*Objectives*. Prehypertension is an early stage of hypertension that is characterized by inflammatory factors. Inflammation also plays an essential role in the development of coronary atherosclerosis (CAS). The present study evaluated the NALP3-inflammasome and its related genes,* NLRP3*,* NOD2*, and* CARD8*, using SNP linkage and gene haplotypes in prehypertensive patients.* Methods*. A total of 576 patients with prehypertension and suspected coronary heart disease (CHD) were enrolled. According to coronary angiography, patients were divided into two groups: arterial stenosis <50% of the diameter (control) and arterial stenosis >50% of the diameter (case). Fifteen polymorphisms in the* NOD2*,* NLRP3*, and* CARD8* genes were analyzed, and serum levels of C-reactive protein (CRP) were measured.* Results*. When comparing allele frequencies, none of these 15 SNPs in* NOD2*,* CARD8*, and* NLPR3* genes showed a significant difference using multiple logistic regression. However, the CTACATAA (*p* = 0.0064) and CCACATAG (*p* = 0.0126) haplotypes of the* NOD2* gene SNPs were significantly different between cases and controls.* Conclusions*. Although our study excludes a significant association of selected SNPs in these genes with CHD in prehypertension patients, this work suggests that the CTACATAA and CCACATAG haplotypes were associated with CHD in the* NOD2* locus. This work suggests that the CTACATAA and CCACATAG haplotypes were associated with CHD in prehypertension patients in the* NOD2* locus.

## 1. Introduction

Hypertension causes approximately 54% of strokes and 47% of coronary heart disease (CHD) cases, and it is a well-established risk factor for coronary atherosclerosis (CAS) [1]. According to the Seventh Report of the Joint National Committee on Prevention, Detection, Evaluation, and Treatment of High Blood Pressure (JNC 7), prehypertension is defined as systolic (S) blood pressure (BP) between 120 mmHg and 139 mmHg and/or diastolic (D) BP between 80 mmHg and 89 mmHg [2]. Many studies have demonstrated that prehypertension increases the likelihood of cardiovascular disease and premature morbidity [3–11]. Furthermore, several large-scale cohort studies have reported that prehypertension is an independent risk factor for stroke and CHD [4, 7, 8, 10]. Arima et al. found that prehypertension and hypertension were predictive of developing CHD in an older population in the Asia-Pacific region [12].

Some of the genetic and pathophysiologic mechanisms that contribute to hypertension also play an important role in prehypertension [13]. Levels of inflammatory factors such as C-reactive protein (CRP) and interleukin-6 (IL-6) are elevated in prehypertensive patients compared with normotensive individuals [14, 15]. Several studies indicate that CRP is a risk factor for CHD, although the association between CRP and CHD was substantially lower after adjustment for conventional cardiovascular risk factors [16–21] As early as 2003, the Centers for Disease Control/American Heart Association guidelines for CRP testing suggested that values >10 mg/L indicate acute inflammation and have uncertain implications for cardiovascular risk prediction [22].

Inflammation and the innate immune system are implicated in the development of CHD [23–25]. In particular, the NOD-like receptor family pyrin domain-containing 3 (*NLRP3*) inflammasome proteins accelerate the progression of CHD. The NALP3 axis comprises apoptosis-associated speck-like protein containing a caspase recruitment domain (ASC), caspase recruitment domain (CARD), and caspase-1 [26, 27]. Following activation of the inflammasome, IL-1 and downstream factors such as IL-6 act as major inflammatory proteins [28]. Therefore, inflammasome-mediated processes are related to the pathophysiology of CAS [29].

Inflammatory signaling is considered to be a salient factor in CAS [30, 31]. An accumulating body of evidence from molecular genetic studies demonstrates that individual genetic background contributes to the development of CHD [32, 33]. Inflammation-related genetic variance has been proposed to increase susceptibility to CHD, but this association has not been confirmed. Single nucleotide polymorphisms (SNPs) have a strong influence on plasma levels and the biological activity of their corresponding proteins [34, 35].

Based on the biologic and pathologic significance of IL-1 [28], this study analyzed SNPs in the* NOD2*,* NLRP3*, and* CARD8* genes in patients with prehypertension. The relationship between SNPs in inflammatory genes and the diagnosis, progression, and prognosis of CHD was investigated.

## 2. Methods

### 2.1. Study Population

A total of 8337 patients presented with prehypertension at the General Hospital of Shenyang Military Region between March 2013 and July 2014. Patients with hypertension or normotension (*n* = 2892) were excluded. All the included patients were aged between 35 and 75 years, with suspected CHD, with/without chest pain, and with/without electrocardiogram (ECG) changes. Patients with chronic kidney disease (CKD), myocardial infarction (MI), coronary revascularization, congenital heart disease, or cerebrovascular diseases were excluded (*n* = 971). Care was taken to eliminate patients receiving drugs that interfere with BP levels (*n* = 3858). Patients that refused to undergo coronary angiography were also excluded (*n* = 40). Data from 576 patients (controls = 281; cases = 295) were finally included. All patients provided written informed consent. The study was conducted in accordance with the Declaration of Helsinki and it was approved by the relevant ethics committee.

### 2.2. Candidate Genes and SNPs Selection

Tag-SNPs selection and genotyping were retrieved from HapMap database for Han population sample using the *r* ^2^-based Tagger program with pairwise *r* ^2^ ≥ 0.80 and MAF ≥ 5%. The selected tag-SNP sites were listed as follows: rs1077861, rs2111235, rs1861759, rs2067085, rs7205423, and rs8056611. The other SNPs were selected a priori based on human gene transcription profiling, pathway analysis, and inflammation associated SNPs reported in the current literature [34, 35]. The total 15 selected SNPs were rs2043211 and rs10403848 in* CARD8;* rs1077861, rs2111235, rs1861759, rs2067085, rs7205423, rs8056611, rs3135499, rs4785225, and rs751271 in* NOD2*; and rs10754558, rs10925027, rs1539019, and rs4612666 in* NLRP3*.

### 2.3. Clinical and Laboratory Test Measurements

Demographic information (age, sex, height, and weight), laboratory examinations, echocardiography, and coronary angiography were obtained from hospital records. BP measurement was performed with an automated aeration device after the patient had rested for more than 5 minutes in a sitting position. The mean BP level was calculated from 3 times of separate measurements, according to the JNC 7 guidelines (SBP ranging from 120 mmHg to 139 mmHg and/or DBP ranging from 80 mmHg to 89 mmHg was categorized as prehypertension). Coronary stenosis was defined by a lumen area >50% in patients with CHD.

Blood samples for SNP analyses were collected at a median time of 12 hours after hospital admission, between 8:00 and 10:00 a.m. the next morning. To standardize blood sampling and to avoid any influence of diurnal variations and food intake, all samples were taken after overnight fast.

DNA was extracted from EDTA-anticoagulated whole blood samples. Genotyping was performed with the polymerase chain reaction (PCR) followed by direct sequencing of the PCR products. Data were generated by Bio Miao Biological Technology (Beijing).

### 2.4. Statistical Analysis

Continuous variables are presented as the mean ± standard deviation (SD) or the median (range) as appropriate. Categorical variables are expressed as percentages (%).Student's *t*-test or Mann-Whitney *U* test was used to compare continuous variables between groups. The *χ* ^2^ test was used to compare the rates of outcomes. Basic data were analyzed using SPSS version 17.0 (SPSS Inc., Chicago, IL). Differences in demographics, variables, and genotypes of the SNPs were evaluated using *χ* ^2^ test. Associations between the fifteen SNPs and CHD risk phenotypes were estimated by calculating odds ratios (ORs) and 95% confidence intervals (CIs) using logistic regression analyses. Bonferroni correction was applied to correct multiple testing. The Hardy-Weinberg equilibrium (HWE) and minor allele frequency (MAF) were determined with Plink software. *p* values <0.05 were considered to denote statistically significant differences between groups. The linkage disequilibrium (LD) block structure was tested by Haploview (version 4.2, Broad Institute, Cambridge, MA) if the MAF was >0.01 and the *p* value of the HWE was >0.05. *D*′ values and *R* ^2^ values for all pairs of SNPs were calculated, and the haplotype blocks were estimated using Haploview.

## 3. Results

### 3.1. Baseline Characteristics of Prehypertensive Patients

A total of 576 prehypertensive patients met the inclusion criteria and underwent selective coronary angiography. The baseline characteristics of patients with and without CHD are shown in Table 1. There were no significant differences between the two cohorts in body mass index, family history of hypertension, uric acid, triglycerides, or platelet count. Older adult patients with prehypertension had more severe coronary stenosis (58.62 ± 7.68 versus 54.34 ± 8.60, *p* < 0.001), while males with prehypertension had a higher risk of CHD (213 versus 132, *p* < 0.001). Patients with more severe stenosis had higher CRP levels (*p* = 0.013) and a lower LVEF (*p* = 0.018). Unhealthy lifestyle habits such as smoking (165 versus 111, *p* < 0.001) and drinking (75 versus 48, *p* = 0.015) were associated with CHD.

### 3.2. Basic Evaluation of the Selected SNPs

None of the 15 SNPs were detected at a frequency of <97%, and the MAFs of these gene SNPs were limited by SNP site selection. Fourteen of these sites displayed good fit with Hardy-Weinberg equilibrium (HWE). While rs2043211 did not fit with HWE, this was possibly because of sample source or sample size, but the reason was not further analyzed. Therefore, this SNP site was not used in subsequent analyses (Table 2). The remaining 14 gene SNP sites had similar frequencies, either alleles or genotypes, to the HapMap gene database (Chinese population; http://hapmap.ncbi.nlm.nih.gov/).

### 3.3. Association between SNPs and Risk of CHD in Prehypertensive Patients

SNPs in the* NLRP3*,* CARD8*, and* NOD2* genes were analyzed in 576 prehypertensive patients (295 cases and 281 controls), all of whom had undergone coronary angiography. One of nine SNPs in* NOD2* was significantly correlated with CHD. However, allele frequencies of the* NLRP3* and* CARD8* SNPs were not significantly different between CHD patients and controls (Tables 3 and 4).

#### 3.3.1. Associations between* NOD2* SNPs and the Risk of CHD in Prehypertensive Patients

All nine of the selected* NOD2* SNP sites were sequenced after PCR, and genotype distributions were determined in prehypertensive patients. The *χ* ^2^ test was used to compare allele and genotype frequencies between CHD patients and controls. Results are presented as ORs according to logistic regression analysis. Among the nine SNP genotypes, three* NOD2* SNPs (rs2111235, rs7205423, and rs8056611) displayed the most significant differences in allele frequency between CHD patients and controls (*p* = 0.004, 0.003, and 0.008, resp.). The frequencies of the TT, TC, and CC genotypes of the* NOD2* gene SNP rs2111235 were 39.32, 49.83, and 10.85%, respectively, in the CHD group and 54.80, 38.08, and 7.12%, respectively, in the control group. Using the TT homozygote genotype as a reference group, the CC genotype was associated with an increased risk of CHD (CC versus TT, OR = 1.458, 95% CI = 1–2.125, *p* = 0.049). But when using Bonferroni correction for multiple comparisons, the associations of rs2111235, rs7205423, and rs8056611 with CHD showed no significance at all. In contrast, there was no relationship between the TC genotype and CHD (TC versus TT, OR = 1.256, 95% CI = 0.771–2.044, *p* = 0.360). In the dominant model, none of the other six polymorphisms exhibited a significant difference in either allele frequency or genotype distributions between CHD cases and controls (Table 3).

#### 3.3.2. Association between the* NLRP3* and* CARD8* SNPs and the Risk of CHD in Prehypertensive Patients

The genotype frequencies of the five* NLRP3* and two* CARD8* SNPs in prehypertensive patients are listed in Table 4. Results are expressed as ORs generated from logistic regression analysis. Comparison of allele and genotype frequencies revealed no significant differences between ACS patients and controls.

#### 3.3.3. Multiple Logistic Regression of Baseline Characteristics and Candidates Gene SNPs

Based on the results above, we used multiple logistic regression of baseline characteristics and possible candidates allele genes (rs2111235, rs7205423, and rs8056611). The results are listed in Table 5. None of these possible genes showed significant differences between normal people and ACS patients (*p* > 0.05).

### 3.4. Disequilibrium and Haplotype Association Analysis of* NOD2* SNPs

**Figure 1:**
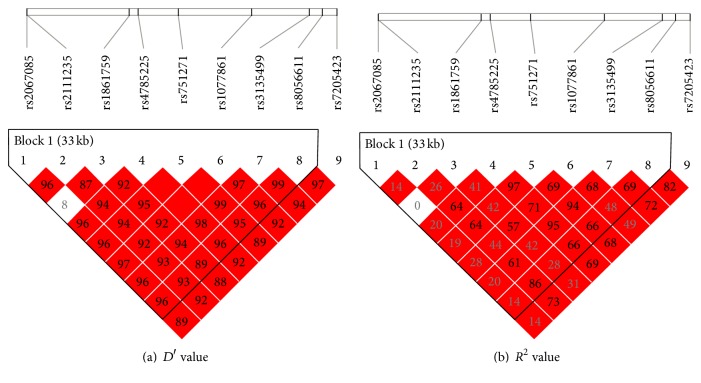
Pairwise *D*′ and *R* ^2^ between selected SNPs. Red squares represent high linkage disequilibrium (LD); white squares represent low LD. *D*′ = 0.96, while *R* ^2^ = 0.14 between SNP1 (rs2067085) and SNP8 (rs4845618).

Among the LD blocks generated from direct sequencing of CHD cases and controls, the tag SNP (rs2067085) was chosen for genotyping by setting the *D*′- and *R* ^2^ value equal to 1. To verify the results, the* NOD2* SNPs were sequentially conditioned on the minor allele of each variant. Results demonstrated that rs1077861, rs2111235, rs1861759, rs2067085, rs8056611, rs3135499, rs4785225, and rs751271 displayed significant LD when analyzed by *D*′ value. When considering *R* ^2^ values, none except for rs8056611 exhibited significant LD between the* NOD2* loci (Figure 1). Thus, rs2111235 and rs8056611 displayed LD between these two SNP sites, enabling the possible haplotype to be inferred for two* NOD2* SNPs. The risk haplotypes CTACATAA (*p* = 0.0064) and CCACATAG (*p* = 0.0126) generated from these six sequences proved to be significantly different between the cases and controls (Table 6).

## 4. Discussion

In this study, patients with more severe coronary stenosis had higher CRP levels and a lower left ventricular ejection fraction (LVEF). Univariate logistic regression analysis of fifteen SNP candidates revealed that rs2111235 in the NOD2 gene was significantly associated with CHD in the prehypertension population. But further multiple regression has negative outcomes where none of these candidate SNP sites may affect the progression of coronary atherosclerosis in prehypertensive people.

Recent research demonstrated that human atherosclerosis is characterized by higher expression of NALP3-inflammasome-related inflammation factors. For example, NOD2, expressed in endothelial cells, macrophages, and endothelial cells, raised in atherosclerotic lesions, was attributed to transcriptional upregulation in the local inflammatory milieu and massive infiltration of macrophages [36]. Compared with the low levels of inflammation factors in healthy arteries, the activity of human atherosclerosis is associated with these factors' enhanced mediated innate immunity. These were also abundantly expressed in human atherosclerosis plaques. NLRP3 was overexpressed in patients with coronary atherosclerosis and this is proved to be correlated with the severity of coronary artery disease and the atherosclerotic risk factors [37]. NOD2 induced cyclooxygenase-2 (COX-2) expression during macrophage activation which is mediated by IL-1*β* and TNF-*α* [36]. However, Paramel et al. have provided the notion that the expression of CARD8 in the atherosclerotic plaque is much higher and the polymorphism of CARD8 gene impacts CARD8 expression levels [38]. Another study found that inflammation factors expression levels were higher in coronary arteries versus healthy controls (heart-beating donors). Cases of macroscopically and microscopically proven CHD showed high serum levels of NALP3-inflammasome-related factors in atherosclerotic plaques, especially in areas infiltrated with inflammatory cells [39].

All these results were based on a serum inflammation index rather than genetics. According to gaps in the current knowledge, we decided to investigate SNPs in genes expressing inflammatory proteins. The selection of SNPs relied on the Chinese genome variation database, and the minor allele frequencies were also matched with the selection requirements.

Scarce research has been conducted on the association between inflammasome-related gene SNPs and coronary lesions in prehypertensive patients. To the authors' knowledge, this is the first study to systematically analyze this relationship. It is well established that NALP3-inflammasome-related factors play an important role in other inflammatory diseases such as Crohn's disease, as indicated by serological indicators and genetics [40, 41]. Although the target disease was different in the present study, there is a large body of literature on NLRP3, NOD2, and CARD8 SNPs and serum expression products and the correlation with inflammasome protein expression and activation. Similar to other inflammatory diseases, CHD has been demonstrated to involve these mechanisms [29].

Prehypertension is a relatively novel concept, which was proposed just over a decade ago, and research concerning inflammatory pathways in prehypertensive patients was limited. Our study was the first one based on the theory that inflammation contributes to the progression of CHD in prehypertensive patients.

We cannot infer that any of these SNP gene site's alleles increases the susceptibility of prehypertension patients to CHD according to this study. But the distribution of the NOD2 SNP sites rs2067085, rs2111235, rs1861759, rs4785225, rs751271, rs1077861, rs3135499, and rs8056611 was consistent with LD including C in rs2067085, T in rs2111235, A in rs1861759, C in rs4785225, A in rs751271, T in rs1077861, A in rs3135499, and A in rs8056611. Tight linkage formed the CTACATAA and the CCACATAG haplotypes which has been proven to have effects on CHD in prehypertension patients. This may be because haplotype-based association method may be more powerful than the single SNP locus test which has already been proven and the messages taken by the haplotype were much larger than single SNP locus [40, 42, 43].

The presence of only a limited number of SNPs in NOD2, NLRP3, and CARD8 in this patient population indicates that these genes are highly conserved. The characteristics associated with SNP sites in these genes in prehypertensive patients have also not been described previously. In this study, we do not find SNPs identified in CHD and non-CHD patients in the Chinese prehypertension population. But we have noticed that the NOD2 gene SNPs rs2067085, rs2111235, rs1861759, rs4785225, rs751271, rs1077861, rs3135499, and rs8056611 exhibited significant LD. The association between NOD2 haplotype and CHD in the prehypertensive population requires further research.

## 5. Limitations

This study has some limitations, including the small cohort size and the recruitment of patients from a single center. It is possible that significant results will be observed in multicenter studies with larger sample sizes. Second, the severity of coronary artery lesions was only evaluated by coronary angiography, which does not produce extensive data on the plaque burden of CHD. Also, the cases and controls were identified according to only one coronary angiography, which might introduce misclassification of cases and controls. Further studies using coronary computed tomography and/or intravascular ultrasound may provide more comprehensive information about the severity of CHD. This study used retrospective data, but more prospective, observational studies are necessary in the future.

## 6. Conclusions

The CTACATAA and CCACATAG haplotypes of the NOD2 SNPs were related to CHD in prehypertensive patients. It was also found that prehypertensive patients with higher serum levels of CRP had a higher risk of CHD.

## Figures and Tables

**Table 1:** Clinical baseline characteristics of prehypertensive patient population.

Variable	Stenosis <50% (*n* = 281)	Stenosis >50% (*n* = 295)	All patients (*n* = 576)	*p* value (<50% versus >50%)
Age (yr)	54.34 ± 8.60	58.62 ± 7.68	56.56 ± 8.40	<0.001
Male	132 (46.98)	213 (72.20)	345 (59.90)	<0.001
BMI (kg/m^2^)	24.77 ± 2.99	25.10 ± 2.80	24.96 ± 2.89	0.292
DM (%)	21 (7.47)	55 (18.64)	76 (13.19)	0.001
Family history of HT (%)	68 (24.20)	65 (22.03)	133 (23.09)	0.538
Family history of DM (%)	56 (19.93)	115 (38.98)	171 (29.69)	<0.001
Serum potassium (mmol/L)	3.99 ± 0.32	4.11 ± 0.38	4.05 ± 0.36	0.002
Serum sodium (mmol/L)	140.83 ± 3.42	140.31 ± 3.15	140.56 ± 3.28	0.125
Urine potassium (mmol/L)	30.07 ± 15.08	29.46 ± 14.06	29.79 ± 14.64	0.686
Urine sodium (mmol/L)	139.74 ± 49.50	121.22 ± 59.51	130.41 ± 70.47	0.011
Uric acid (mmol/L)	296.99 ± 91.73	304.97 ± 75.39	302.11 ± 85.79	0.357
CRP	2.47 ± 4.01	3.52 ± 4.14	3.02 ± 4.10	0.013
LDL-C (mmol/L)	2.67 ± 0.89	2.30 ± 0.93	2.48 ± 0.93	<0.001
TG (mmol/L)	1.85 ± 1.54	1.96 ± 1.13	1.91 ± 1.34	0.457
PLT (10^9^/L)	215.82 ± 51.62	218.33 ± 58.36	216.95 ± 55.20	0.660
LVEF (%)	66.60 ± 4.91	64.90 ± 6.24	65.60 ± 5.96	0.018
Smoking (%)	111 (39.57)	165 (55.83)	276 (47.92)	0.001
Alcohol (%)	48 (17.08)	75 (25.42)	123 (21.35)	0.015

BMI: body mass index; DM: diabetes mellitus; HT: hypertension; CRP: C-reactive protein; LDL: low-density lipoprotein; TG: triglycerides; PLT: platelet count; LVEF: left ventricular ejection fraction.

**Table 2:** Gene SNPs' Hardy-Weinberg equilibrium and MAF among prehypertensive patients.

Genotype SNPs		HWE			MAF	Detectability	Detectable rate (%)
		O(HET)	E(HET)	*p* value^*∗*^			
NOD2	rs1077861	0.297	0.306	0.613	0.188	566	98.26
	rs1861759	0.238	0.235	1.000	0.135	561	97.40
	rs2067085	0.111	0.124	0.067	0.066	567	98.44
	rs2111235	0.44	0.428	0.632	0.311	562	97.57
	rs3135499	0.348	0.368	0.326	0.243	566	98.26
	rs4785225	0.348	0.371	0.265	0.246	564	97.92
	rs7205423	0.398	0.407	0.704	0.284	566	98.26
	rs751271	0.352	0.375	0.220	0.25	567	98.44
	rs8056611	0.431	0.429	1.000	0.311	564	97.92

CARD8	rs2043211	0.555	0.498	0.038	0.472	564	97.92
	rs10405768	0.428	0.428	1.000	0.310	562	97.57

NLRP3	rs4612666	0.504	0.490	0.674	0.430	566	98.26
	rs1539019	0.544	0.499	0.098	0.474	566	98.26
	rs10754558	0.479	0.500	0.410	0.495	564	97.92
	rs10925027	0.489	0.499	0.756	0.473	564	97.92

HWE: Hardy-Weinberg equilibrium; O(HET): observed heterozygosity; E(HET): expected heterozygosity; MAF: minor allele frequency. ^*∗*^ *p* value > 0.05, fit to HWE.

**Table 3:** Genotypes and allele frequencies of the *NOD2* gene SNP polymorphisms among prehypertensive patients.

Genotype SNPs	Allele		Genotype (stenosis < 50%)			Genotype (stenosis ≥ 50%)			Allele A versus B				AA versus BB			BB versus AB		
	A^*∗*^	B	AA (%)	AB (%)	BB (%)	AA (%)	AB (%)	BB (%)	*χ* ^2^	OR (95% CI)	*p* value	*Bonf-p*	OR (95% CI)	*p* value	*Bonf-p*	OR (95% CI)	*p* value	*Bonf-p*
rs1077861	A	T	197 (70.11)	77 (27.40)	7 (2.49)	185 (62.71)	94 (31.86)	16 (5.42)	2.769	1.367 (0.945–1.976)	0.096	1.000	1.449 (0.835–2.513)	0.187	1.000	0.8981 (0.4619–1.746)	0.187	1.000
rs1861759	A	C	221 (78.65)	57 (20.28)	3 (1.07)	208 (70.51)	80 (27.12)	7 (2.37)	3.247	1.477 (0.965–2.260)	0.072	1.000	1.461 (0.620–3.44)	0.386	1.000	1.026 (0.395–2.667)	0.957	1.000
rs2067085	C	G	249 (88.61)	32 (11.39)	0 (0)	257 (87.12)	32 (10.85)	6 (2.03)	0.956	1.335 (0.747–2.386)	0.328	1.000	<0.001	0.999	1.000	<0.001	0.999	1.000
rs2111235	T	C	154 (54.80)	107 (38.08)	20 (7.12)	116 (39.32)	147 (49.83)	32 (10.85)	8.15	1.576 (1.152–2.155)	0.004	0.006	1.458 (1–2.125)	0.049	0.735	1.256 (0.771–2.044)	0.360	1.000
rs3135499	A	C	176 (62.63)	89 (31.67)	16 (5.69)	160 (54.24)	112 (37.97)	23 (7.80)	2.506	1.310 (0.937–1.832)	0.113	1.000	1.222 (0.810–1.843)	0.339	1.000	1.128 (0.660–1.929)	0.660	1.000
rs4785225	C	G	173 (61.57)	91 (32.38)	17 (6.05)	160 (54.24)	110 (37.29)	25 (8.47)	2.345	1.298 (0.929–1.812)	0.126	1.000	1.251 (0.834–1.878)	0.280	1.000	1.05 (0.616–1.788)	0.858	1.000
rs7205423	C	G	168 (59.79)	95 (33.81)	18 (6.41)	130 (44.07)	135 (45.76)	30 (10.17)	8.885	1.627 (1.180–2.243)	0.003	0.045	1.469 (1–2.158)	0.050	0.750	1.251 (0.756–2.07)	0.576	1.000
rs751271	A	C	174 (61.92)	89 (31.67)	18 (6.41)	157 (53.22)	114 (38.64)	24 (8.14)	2.824	1.328 (0.953–1.850)	0.093	1.000	1.215 (0.817–1.807)	0.337	1.000	1.178 (0.697–1.991)	0.540	1.000
rs8056611	A	G	153 (54.45)	106 (37.72)	22 (7.83)	119 (40.34)	142 (48.14)	34 (11.53)	7.083	1.526 (1.117–2.085)	0.008	0.120	1.419 (0.984–2.047)	0.061	0.915	1.217 (0.753–1.969)	0.423	1.000

SNP: single nucleotide polymorphism; CI: confidence interval; OR: odds ratio.

*Bonf-p*: *p* value with Bonferroni correction.

^*∗*^Allele A is the wild-type allele.

**Table 4:** Genotypes and allele frequencies of *NLRP3 *and *CARD8* SNP polymorphisms among prehypertensive patients.

Genotype SNPs		Allele		Genotype (stenosis < 50%)			Genotype (stenosis ≥ 50%)			Allele A versus B				AA versus BB			BB versus AB		
		A^*∗*^	B	AA (%)	AB (%)	BB (%)	AA (%)	AB (%)	BB (%)	*χ* ^2^	OR (95% CI)	*p* value	*Bonf-p*	OR (95% CI)	*p* value	*Bonf-p*	OR (95% CI)	*p* value	*Bonf-p*
CARD8	rs10403848	G	A	139 (49.47)	114 (40.57)	28 (9.96)	135 (45.76)	132 (44.75)	28 (9.49)	0.193	1.072 (0.787–1.461)	0.661	1.000	1.006 (0.703–1.438)	0.976	1.000	1.182 (0.736–1.898)	0.488	1.000

NLRP3	rs4612666	C	T	86 (30.61)	145 (51.60)	50 (17.80)	98 (33.22)	145 (49.15)	52 (17.63)	0.178	0.780 (0.585–1.038)	0.673	1.000	0.950 (0.704–1.281)	0.734	1.000	0.922 (0.610–1.392)	0.698	1.000
	rs1539019	C	T	68 (24.20)	143 (50.89)	70 (24.91)	79 (26.78)	170 (57.63)	46 (15.59)	2.920	1.576 (1.152–2.155)	0.090	1.000	0.743 (0.547–1.008)	0.056	0.840	1.372 (0.910–2.039)	0.132	0.588
	rs10754558	C	G	63 (22.42)	138 (49.11)	80 (28.47)	90 (30.51)	138 (46.78)	67 (22.71)	3.569	0.759 (0.570–1.011)	0.060	0.900	0.767 (0.580–1.017)	0.066	0.990	0.906 (0.603–1.361)	0.635	1.000
	rs10925027	C	T	92 (32.74)	131 (46.62)	58 (20.64)	70 (23.73)	151 (51.19)	74 (25.08)	3.212	1.3 (0.976–1.732)	0.073	1.000	1.284 (0.964–1.710)	0.088	1.000	1.184 (0.787–1.779)	0.418	1.000

SNP: single nucleotide polymorphism; CI: confidence interval; OR: odds ratio.

*Bonf-p*: *p* value with Bonferroni correction.

^*∗*^Allele A is the wild-type allele.

**Table 5:** Multiple logistic regression of baseline characteristics and candidates allele genes.

Variable	Univariate analysis	Multivariate analysis		
	*p* value	OR	95% CI	*p* value
Gender	0.000	6.157	2.790–13.584	0.000
Age	0.000	1.071	1.030–1.113	0.001
Family history of HT	0.657	0.705	0.333–1.491	0.360
DM	0.040	3.547	1.535–8.197	0.003
Family history of CHD	0.000	5.029	2.676–9.451	0.000
SBP	0.000	1.065	1.029–1.103	0.000
Urine sodium	0.087	0.997	0.992–1.001	0.092
Serum potassium	0.031	2.308	0.969–5.496	0.059
LDL-C	0.768	0.839	0.630–1.118	0.231
Smoking	0.112	0.756	0.354–1.613	0.469
Alcohol	0.098	1.280	0.620–2.643	0.504
rs2111235	0.043	0.201	0.017–2.326	0.199
rs10403848	0.031	0.907	0.351–2.345	0.840
rs10754558	0.050	1.543	0.700–3.402	0.282
rs1077861	0.133	1.352	0.716–2.553	0.353
rs10925027	0.087	0.580	0.291–1.157	0.122
rs1539019	0.191	1.289	0.565–2.939	0.546
rs1861759	0.061	0.097	0.005–1.867	0.122
rs2067085	0.109	0.519	0.214–2.657	0.495
rs3135499	0.233	1.913	0.026–140.544	0.767
rs4612666	0.074	1.241	0.601–2.564	0.559
rs7205423	0.263	1.465	0.134–16.034	0.754
rs751271	0.132	3.283	0.072–150.134	0.542
rs8056611	0.098	1.501	0.109–20.728	0.762
rs4785225	0.061	0.892	0.655–1.324	0.183
rs10405768	0.078	0.679	0.312–1.569	0.514

**Table 6:** Haplotype association with *NOD2* locus in prehypertensive patients.

Haplotype	Frequency	Case, control frequencies	*χ* ^2^	*p* value
H1: CTACATAA	0.666	0.621, 0.714	7.437	0.0064
H2: CCCGCACG	0.108	0.119, 0.098	0.839	0.3596
H3: CCACATAG	0.063	0.085, 0.041	6.227	0.0126
H4: CCAGCTCG	0.056	0.057, 0.054	0.02	0.8877
H5: GCAGCACG	0.051	0.054, 0.049	0.127	0.722
H6: GCCGCACG	0.011	0.015, 0.006	1.656	0.1981

Haplotypes had plausible association with CHD in prehypertensive patients.

## References

[1] Chobanian A. V., Bakris G. L., Black H. R. (2003). The Seventh report of the joint national committee on prevention, detection, evaluation, and treatment of high blood pressure: the JNC 7 report. *The Journal of the American Medical Association*.

[2] Lawes C. M., Hoorn S. V., Rodgers A. (2008). Global burden of blood-pressure-related disease, 2001. *The Lancet*.

[3] Asia Pacific Cohort Studies Collaboration (2003). Blood pressure indices and cardiovascular disease in the Asia Pacific region: a pooled analysis. *Hypertension*.

[4] He J., Gu D., Chen J. (2009). Premature deaths attributable to blood pressure in China: a prospective cohort study. *The Lancet*.

[5] Vasan R. S., Larson M. G., Leip E. P. (2001). Impact of high-normal blood pressure on the risk of cardiovascular disease. *The New England Journal of Medicine*.

[6] Liszka H. A., Mainous A. G., King D. E., Everett C. J., Egan B. M. (2005). Prehypertension and cardiovascular morbidity. *Annals of Family Medicine*.

[7] Hsia J., Margolis K. L., Eaton C. B. (2007). Prehypertension and cardiovascular disease risk in the women's health initiative. *Circulation*.

[8] Conen D., Ridker P. M., Buring J. E., Glynn R. J. (2007). Risk of cardiovascular events among women with high normal blood pressure or blood pressure progression: prospective cohort study. *British Medical Journal*.

[9] Murakami Y., Hozawa A., Okamura T., Ueshima H. (2008). Relation of blood pressure and all-cause mortality in 180,000 Japanese participants: pooled analysis of 13 cohort studies. *Hypertension*.

[10] Asayama K., Ohkubo T., Yoshida S. (2009). Stroke risk and antihypertensive drug treatment in the general population: the Japan arteriosclerosis longitudinal study. *Journal of Hypertension*.

[11] Ishikawa Y., Ishikawa J., Ishikawa S. (2010). Prehypertension and the risk for cardiovascular disease in the Japanese general population: the Jichi Medical School Cohort Study. *Journal of Hypertension*.

[12] Arima H., Murakami Y., Lam T. H. (2012). Effects of prehypertension and hypertension subtype on cardiovascular disease in the Asia-Pacific region. *Hypertension*.

[13] Drukteinis J. S., Roman M. J., Fabsitz R. R. (2007). Cardiac and systemic hemodynamic characteristics of hypertension and prehypertension in adolescents and young adults: The Strong Heart Study. *Circulation*.

[14] King D. E., Egan B. M., Mainous A. G., Geesey M. E. (2004). Elevation of C-reactive protein in people with prehypertension. *Journal of Clinical Hypertension*.

[15] Chrysohoou C., Pitsavos C., Panagiotakos D. B., Skoumas J., Stefanadis C. (2004). Association between prehypertension status and inflammatory markers related to atherosclerotic disease: the ATTICA study. *American Journal of Hypertension*.

[16] Jager A., van Hinsbergh V. W. M., Kostense P. J. (1999). von Willebrand factor, C-reactive protein, and 5-year mortality in diabetic and nondiabetic subjects: the Hoorn Study. *Arteriosclerosis, Thrombosis, and Vascular Biology*.

[17] Mendall M. A., Strachan D. P., Butland B. K. (2000). C-reactive protein: relation to total mortality, cardiovascular mortality and cardiovascular risk factors in men. *European Heart Journal*.

[18] Doggen C. J. M., Berckmans R. J., Sturk A., Manger Cats V., Rosendaal F. R. (2000). C-reactive protein, cardiovascular risk factors and the association with myocardial infarction in men. *Journal of Internal Medicine*.

[19] Gram J., Bladbjerg E.-M., Møller L., Sjøl A., Jespersen J. (2000). Tissue-type plasminogen activator and C-reactive protein in acute coronary heart disease. A nested case-control study. *Journal of Internal Medicine*.

[20] Packard C. J., O'Reilly D. S. J., Caslake M. J. (2000). Lipoprotein-associated phospholipase A2 as an independent predictor of coronary heart disease. *The New England Journal of Medicine*.

[21] Folsom A. R., Aleksic N., Catellier D., Juneja H. S., Wu K. K. (2002). C-reactive protein and incident coronary heart disease in the Atherosclerosis Risk in Communities (ARIC) study. *American Heart Journal*.

[22] Pearson T. A., Mensah G. A., Alexander R. W. (2003). Markers of inflammation and cardiovascular disease: application to clinical and Public Health practice: a statement for healthcare professionals from the Centers for Disease Control and Prevention and the American Heart Association. *Circulation*.

[23] Hansson G. K., Hermansson A. (2011). The immune system in atherosclerosis. *Nature Immunology*.

[24] Libby P., Ridker P. M., Hansson G. K. (2011). Progress and challenges in translating the biology of atherosclerosis. *Nature*.

[25] Weber C., Noels H. (2011). Atherosclerosis: current pathogenesis and therapeutic options. *Nature Medicine*.

[26] Martinon F., Burns K., Tschopp J. (2002). The Inflammasome: a molecular platform triggering activation of inflammatory caspases and processing of proIL-*β*. *Molecular Cell*.

[27] Yajima N., Takahashi M., Morimoto H. (2008). Critical role of bone marrow apoptosis-associated speck-like protein, an inflammasome adaptor molecule, in neointimal formation after vascular injury in mice. *Circulation*.

[28] Dinarello C. A. (1994). The interleukin-1 family: 10 years of discovery. *FASEB Journal*.

[29] Lu X., Kakkar V. (2014). Inflammasome and atherogenesis. *Current Pharmaceutical Design*.

[30] Hansson G. K. (2005). Mechanisms of disease: inflammation, atherosclerosis, and coronary artery disease. *The New England Journal of Medicine*.

[31] Harismendy O., Notani D., Song X. (2011). 9p21 DNA variants associated with coronary artery disease impair interferon-*γ* signalling response. *Nature*.

[32] McPherson R., Davies R. W. (2012). Inflammation and coronary artery disease: insights from genetic studies. *Canadian Journal of Cardiology*.

[33] Deloukas P., Kanoni S., Willenborg C. (2013). Large-scale association analysis identifies new risk loci for coronary artery disease. *Nature Genetics*.

[34] Tomic V., Russwurm S., Möller E. (2005). Transcriptomic and proteomic patterns of systemic inflammation in on-pump and off-pump coronary artery bypass grafting. *Circulation*.

[35] Calvano S. E., Xiao W., Richards D. R. (2005). A network-based analysis of systemic inflammation in humans. *Nature*.

[36] Liu H.-Q., Zhang X.-Y., Edfeldt K. (2013). NOD2-mediated innate immune signaling regulates the eicosanoids in atherosclerosis. *Arteriosclerosis, Thrombosis, and Vascular Biology*.

[37] Zheng F., Xing S., Gong Z., Xing Q. (2013). NLRP3 inflammasomes show high expression in Aorta of patients with atherosclerosis. *Heart Lung and Circulation*.

[38] Paramel G. V., Folkersen L., Strawbridge R. J. (2013). CARD8 gene encoding a protein of innate immunity is expressed in human atherosclerosis and associated with markers of inflammation. *Clinical Science*.

[39] El Mokhtari N. E., Ott S. J., Nebel A. (2007). Role of NOD2/*CARD15* in coronary heart disease. *BMC Genetics*.

[40] Xu Z., Kaplan N. L., Taylor J. A. (2007). Tag SNP selection for candidate gene association studies using HapMap and gene resequencing data. *European Journal of Human Genetics*.

[41] Carlson C. S., Eberle M. A., Rieder M. J., Yi Q., Kruglyak L., Nickerson D. A. (2004). Selecting a maximally informative set of single-nucleotide polymorphisms for association analyses using linkage disequilibrium. *American Journal of Human Genetics*.

[42] Carlson C. S., Eberle M. A., Rieder M. J., Yi Q., Kruglyak L., Nickerson D. A. (2004). Selecting a maximally informative set of single-nucleotide polymorphisms for association analyses using linkage disequilibrium. *The American Journal of Human Genetics*.

[43] Xu Z., Taylor J. A. (2009). SNPinfo: integrating GWAS and candidate gene information into functional SNP selection for genetic association studies. *Nucleic Acids Research*.

